# The Effect of Cementitious Materials on the Engineering Properties and Pore Structure of Concrete with Recycled Fine Aggregate

**DOI:** 10.3390/ma16010305

**Published:** 2022-12-28

**Authors:** Zihao Liu, Koji Takasu, Hiroki Suyama, Hidehiro Koyamada, Shilun Liu, Qi Hao

**Affiliations:** 1 Architecture Course, Graduate School of Environmental Engineering, The University of Kitakyushu, 1-1 Hibikino Wakamatsu, Kitakyushu, Fukuoka 8080135, Japan; 2 Department of Architecture, Faculty of Environmental Engineering, The University of Kitakyushu, 1-1 Hibikino Wakamatsu, Kitakyushu, Fukuoka 8080135, Japan

**Keywords:** compressive strength, drying shrinkage, carbonation, pore structure, cementitious materials, recycled fine aggregate

## Abstract

With the rapid development of urbanization, the construction industry consumes a lot of cement and produces a large amount of construction waste. To overcome this situation, the rational use of recycled aggregate produced from waste concrete is one of solutions. In some countries, the building industry has approved the use of recycled coarse aggregates in concrete, with some limits. However, practically all existing standards and regulations prohibit the use of recycled fine aggregate (RFA) in concrete. Therefore, study on improving the performance of RFA concrete is vital. In this study, the effects of fly ash and GGBS on concrete with RFA were investigated. Compressive strength, pore structure, drying shrinkage and accelerated carbonation were tested. The correlation between the pore structure and properties of concrete was analyzed. The results show that adding fly ash and GGBS to RFA concrete increased its compressive strength, modified pore structure, reduced drying shrinkage, and even achieved higher compressive strength and lower drying shrinkage than normal concrete. The compressive strength was mainly affected by the capillary pores, and the carbonation was mainly affected by the gel pores.

## 1. Introduction

Since the beginning of the 21st century, concrete has been a widely used building material, with yearly production estimated at 10 billion m^3^ [1]. Aggregate includes coarse aggregate (gravel) and fine aggregate (sand), accounting for about 60–80% of the total volume of concrete [2]. Because of the extensive use of concrete, the demand for aggregates has greatly increased [3], and the production of fine aggregates by crushing gravel has a high energy cost and causes problems with fresh concrete because of high angularity [4,5]. However, the demolition of old buildings generates a large amount of construction waste, at 850 to 880 Mt/year in the European Union [6,7], 317 Mt/year in the US, and 77 Mt/year in Japan [8]. Therefore, the rational use of recycled aggregate produced from waste concrete can simultaneously solve problems with building material supply and disposal. The building industry has previously approved the use of recycled coarse aggregates in concrete, with some limits, and in some countries, full substitution is permitted in certain circumstances. However, practically all existing standards and regulations prohibit the use of recycled fine aggregate (RFA) in the manufacturing of concrete and mortar [9].

Several studies have been conducted on the properties of RFA concrete. Gholampour et al. [10] studied concrete containing 25, 50, and 100% RFA, and their results showed that the compressive strength of the concrete decreased as the replacement rate of RFA increased, although the strength of concrete containing 25% RFA was slightly increased. Kirthik et al. [11] showed that increasing the content of RFA in concrete decreased its durability, and the best RFA content was 30%, which decreased shrinkage and porosity by 14% and 25%, respectively, and increased resistance to chlorine penetration by 21%. Khatib et al. [12] studied concrete containing 0, 25, 50, and 100% RFA, and showed that concrete containing 100% and 25% RFA had 30% and 15% lower compressive strength, respectively, compared with normal concrete, and increasing the RFA content increased shrinkage. The chloride permeability of concrete increases with the RFA content, whereas incorporating fly ash decreases chloride permeability [13,14,15,16,17]. Lovato et al. [18] reported that the carbonation depth of concrete increased with the content of RFA. Evangelista et al. [19] showed that in concrete containing 30% and 100% RFA, the carbonation depth increased by 40% and 100%, respectively, compared with normal concrete. Bu et al. [20] reviewed the literature on the durability of concrete containing RFA and found that the durability of concrete decreased as the replacement rate of RFA increased; for concrete containing 100% RFA, the drying shrinkage of was twice that of ordinary concrete and the carbonation depth increased by about 110%. The density and mechanical properties of concrete decreases as the RFA content increases [21,22]. In summary, exceeding an RFA content in concrete of 30% has several negative effects, including increased shrinkage, decreased compressive strength, decreased carbonation resistance, and increased water absorption. This has greatly limited the application of RFA in concrete, so finding a method that can modify the properties of RFA concrete should expand its applications in practical engineering.

On the other hand, some studies have shown that the application of cementitious materials in concrete can improve durability, reduce long-term deformation, and modify the pore structure. It has been reported that cementitious materials enhance the workability, improve the performance of concrete at high temperatures, and inhibit the alkali-aggregate reaction [23]. Fly ash can be used instead of OPC to decrease porosity and reduce average pore size. Additionally, the volume of the gel’s pores (less than 0.01 μm) increases with the fly ash content [24]. The fly ash content in concrete is about 20%, and its compressive strength is the largest [25]. Adding fly ash to concrete reduces porosity and changes water absorptivity and chloride permeability [26]. High replacement volumes of fly ash increase resistance to chloride penetration substantially [27]. A fly ash content of 50% in concrete mixes offers benefits such as high resistance to chloride and sulfate attack, reduced alkali-silica expansion, and low heat generation [28]. The addition of fly ash to concrete can also reduce the creep [29,30] and drying shrinkage of concrete [26,31]. Özbay et al. [32] found that using GGBS in concrete increases the long-term mechanical properties of concrete. Additionally, using GGBS improves the deformation of concrete and increases the durability of concrete. Adding GGBS to concrete can reduce the porosity and modify the pore structure of the concrete [33]. Concrete containing GGBS tends to have lower shrinkage and creep than ordinary concrete [34,35,36,37]. In addition, some scholars have studied the use of cementitious materials in recycled aggregate concrete (fine and coarse). Qureshi et al. [38] studied recycled coarse aggregate concrete containing 20% fly ash and 30% GGBS, and their results showed that the concrete containing 20% fly ash and 30% GGBS had 2–10% and 5–12% higher compressive strength, respectively. Ahmad et al. [39] reported that the addition of GGBS to recycled coarse aggregate concrete can significantly increase its slump and strength. Kurad et al. [40] analyzed the effect of fly ash on concrete containing recycled fine and coarse aggregate and showed that the concrete had lower initial strength, but fly ash had little effect on its the strength. Ali et al. [41] showed that recycled coarse aggregate concrete containing 20–40% fly ash had a higher compressive strength at 180 days than control concrete. It has also been reported that fly ash improves mechanical properties and significantly reduces water absorption and chloride penetration of recycled coarse aggregate concrete [42]. Kou et al. [43] reported that GGBS and FA had great contributions to the performance of recycled coarse aggregate concrete. Anastasiou et al. [44] studied RFA concrete containing fly ash and showed that fly ash improves long-term strength and decreases water penetration under pressure, and chloride ion penetration. In conclusion, using cementitious materials such as fly ash or GGBS in concrete can improve durability, reduce long-term deformation, and modify pore structure. However, most researchers paid attention to recycled coarse aggregate. Research on the effect of cementitious materials on RFA concrete is still insufficient and requires further investigation. In addition, studies on the effect of GGBS and fly ash have mainly focused on mechanical properties and durability, and little research has been done on microscopic pore structure.

The purpose of this study was to enhance the compressive strength and durability performance of RFA concrete by adding cementitious materials, and to analyze the influence of microstructure on the mechanical properties and durability of RFA concrete. The optimal addition rate of fly ash or GGBS in RFA concrete was obtained. After adding supplementary cementitious materials, RFA concrete had higher strength and lower drying shrinkage than ordinary concrete. In addition, the influence of pore volume of different pore diameters on the compressive strength and durability of RFA concrete was analyzed. The micropores in RFA concrete were divided into harmful pores, small harmful pores, and harmless pores according to diameter. The results of this study will provide a basis for the application of RFA in practical engineering.

## 2. Materials and Experimental Program

### 2.1. Materials Properties

Sea sand (S) and M standard RFA conforming to JIS A 5022 [45] were used as fine aggregate. RFA was made from waste concrete after crushing, grinding and classifying. Crushed stone aggregate was used for coarse aggregate (G). Table 1 shows the physical properties of the fine and coarse aggregates.

OPC as defined in JIS R 5210 [46], supplementary cementitious materials were fly ash (FA) conforming to Class II in JIS A 6201 [47], fly ash with a higher carbon content from a local power plant that had its carbon content reduced by the floatation method (modified fly ash [MFA]), and GGBS as defined in JIS A 6206 [48]. The properties of cement, FA, MFA, and GGBS are shown in Table 2.

### 2.2. Mix Proportions

The mix proportions of the concrete cast in this experiment are shown in Table 3. The design strength of concrete was 27 MPa, according to JASS 5 [49], water-binder ratio was set to 0.55, the unit water volume was 180 kg/m^3^, and the unit coarse aggregate amount was 945 kg/m^3^. A total of 12 concrete mixes were prepared: the control concrete, concrete containing sea sand replaced with RFA (50% by volume), four mixes containing cement replaced with FA or MFA (10% and 15% by weight), two mixes containing cement replaced with GGBS (30% and 45% by weight), and four mixes with cement replaced (30% and 45% by weight) by FA and GGBS using ternary binders (the ratio of FA to GGBS was 1 or 0.5). In the process of concrete production, the aggregates were all surface dry. 

### 2.3. Experiment Method

According to JIS A 1108 [50], testing for compressive strength was performed on cylindrical specimens that were 100 mm in diameter and 200 mm in height. The specimens were cast in a mold and kept for 24 h in a room at 20 °C and 60% RH before demolding. The specimens were then cured in water at 20 °C until the age required for the test. The compressive strength was tested at 7, 28, and 91 days. In order to avoid impact loads on concrete specimens, the loading rate was set at 0.6 ± 0.4 MPa per second. Three specimens were tested for each group, and the average value was taken.

The “Method of measurement for length change of mortar and concrete” described in JIS A 1129-2 [51] was used to conduct the drying shrinkage test, and prismatic specimens measuring 100 × 100 × 400 mm were cast. The specimens were demolded 1 day after casting, and then cured in water at 20 °C for 7 days. After 7 days, a stainless-steel chip was attached to both ends of the specimens, and the length of the specimen was measured as the base length. The specimens were then cured in a constant humidity and temperature chamber (20 ± 1.0 °C; RH, 60 ± 5%) and tested after 182 days.

The accelerated carbonation experiment was conducted according to JIS A1153 [52] using 40 × 40 × 160 mm test specimens. The specimens were cured in water at 20 °C for 4 weeks, and then placed in a thermo-hygrostat at 20 °C and 60% relative humidity for 4 weeks. After curing, the specimens were placed in a carbonation chamber at a CO_2_ concentration of 2.0%, 20 °C, and RH of 65% for 7, 28, 56, and 91 days. The depth of carbonation was tested at a specified age by splitting the specimen at right angles to the length direction and immediately spraying the split surface with 1% phenolphthalein solution to measure the depth-stained red purple. The carbonation depth of was measured at five points on each side (total of 10 points), and the average was taken as the carbonation depth. The carbonation depth was modelled by Equation (1):

Xc = K√t (1)

where Xc is carbonation depth (mm), t is carbonation age (weeks), and K is the carbonation coefficient (mm/weeks ^0.5^).

Porosity was measured by mercury intrusion porosimetry (MIP). The samples were prepared by crushing a specimen (φ100 × 200 mm) that had been cured in water at 20 °C to the specific material age and sieving the powder to obtain particles of 2.5 to 5.0 mm. The hydration reaction was stopped by immersion in acetone, and then the powder was dried under vacuum for 72 h before use. The porosity was tested at 7, 28, and 91 days to investigate the development of the pore structure of concrete.

## 3. Results and Discussion

### 3.1. Compressive Strength

**Figure 1:**
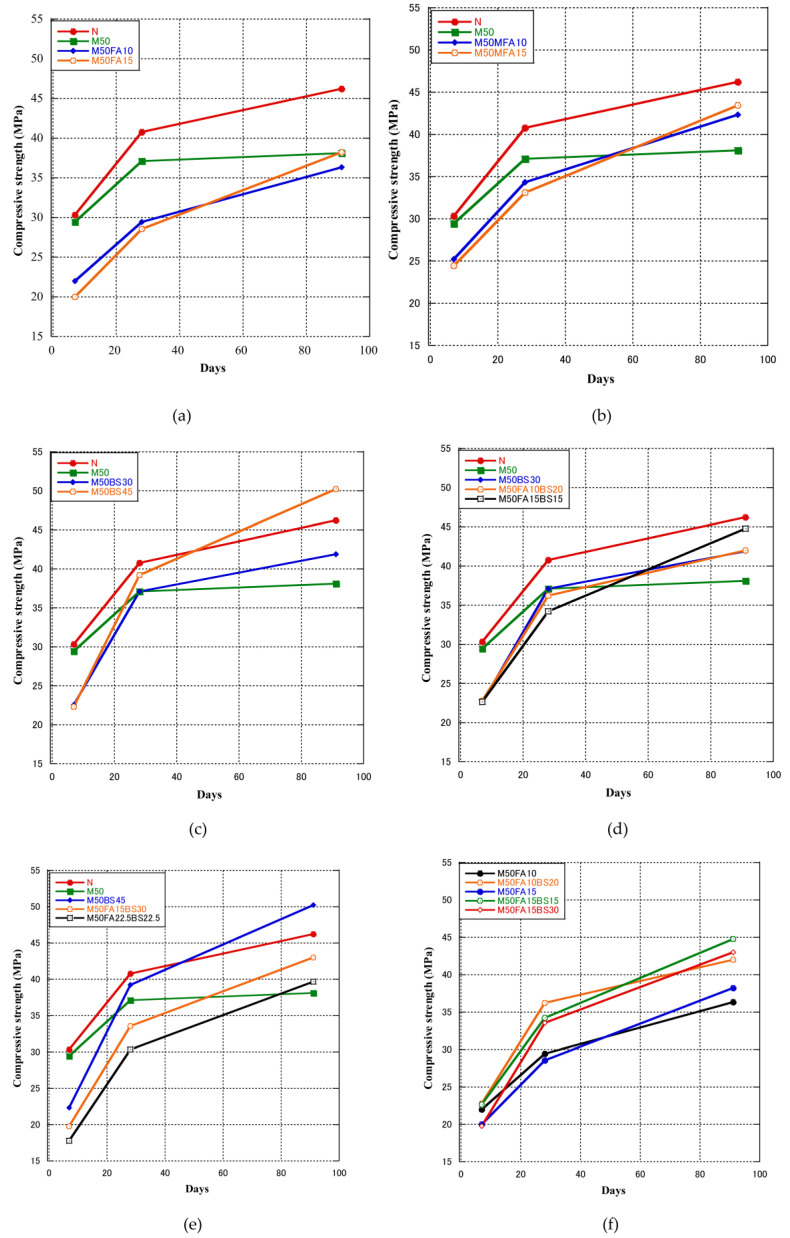
Compressive strength of concrete with cementitious materials.

Figure 1a,b shows the compressive strength of RFA concrete containing only FA or MFA. The concrete containing 50% RFA had lower compressive strength than the control concrete. Compared with the control concrete, the compressive strength at 7, 28, and 91 days decreased by 2.8, 8.9, and 17.6%, respectively. Khatib [12] reported that when sand was substituted with RFA, the long-term strength was systematically decreased. At a replacement level of 100%, this reduction might approach 30%. A reduction of only 15% resulted from a replacement level of 25%. This was attributable to RFA’s porous structure and higher water absorption [24]. The FA specimens, consisting of RFA concrete containing FA, had a lower compressive strength than the M50 concrete at 7 and 28 days, and the MFA specimens showed similar results. The compressive strength of the concrete containing 15% FA was lower than that of the concrete containing 10% FA at 7 and 28 days. However, the decrease of compressive strength at 91 days was not significant. These decreases can be attributed to fly ash’s diluting effect and early-stage poor reactivity [53,54,55,56,57]. The results are in good agreement with the results of some previous studies, which showed that the long-term mechanical characteristics of fly ash concrete had clearly improved [54,58,59,60]. The RFA concrete specimens containing MFA had a higher 91-day compressive strength than the M50 concrete. Compared with M50 concrete, the compressive strength of MFA10 and MFA15 concrete at 91 days increased by 10.93% and 14.04%, respectively. Therefore, the removal of unburned carbon from fly ash by flotation is an effective method for using fly ash with high carbon content in concrete. As shown in Table 2, MFA had a larger specific surface area than ordinary fly ash, which may be an important reason for the better performance of MFA than ordinary fly ash. In addition, the 91-day compressive strength of M50FA10 concrete was 4.8% lower than that of M50 concrete, whereas that of M50FA15 concrete was similar. According to previous studies, the results showed that the reaction degree of fly ash varied depending on the fly ash properties, from less than 4% at 3 days to 9–23% at 28 days to 26–33% at 180 days [53,61,62,63,64].

The pozzolanic reaction between fly ash and Ca(OH)_2_ might result in abundant CSH, resulting in modified pore structure and improved long-term strength, which is ultimately responsible for the improvement in the mechanical performance [56,63,65]. The results of pore structure in this experiment also indicated the pozzolanic reaction.

Figure 1c shows the compressive strength of RFA concrete containing only GGBS. The compressive strengths of M50BS30 and M50BS45 were lower than that of the M50 and control concrete at 7 days. The 28-day compressive strengths of M50BS30 and M50BS45 were higher than that of M50, but lower than that of the control concrete. Both M50BS30 and M50BS45 had a higher 91-day compressive strength than M50, and M50BS45 had a higher 91-day compressive strength than the control concrete. Therefore, using GGBS in RFA concrete decreased the 7-day compressive strength, but did not affect the 28-day compressive strength. In addition, GGBS increased the 91-day compressive strength, and the compressive strength increased with the GGBS content. Some studies also reported similar results [37,66,67]; the early strength of GGBS concrete was lower, and with the increase of curing time, the compressive strength of GGBS concrete increased faster than that of the normal concrete. The compressive strength increases of GGBS concrete took longer due to the slow pozzolanic reaction [66,68]. As shown in Table 2, GGBS has a larger specific surface area and higher CaO content than fly ash. The GGBS group exhibited higher compressive strength than the fly ash group due to the higher CaO content and larger specific surface area of GGBS, which resulted in a better pozzolanic reaction rate in GGBS-based concrete.

Figure 1d shows that the compressive strength of RFA concrete containing 30% blended cementitious materials was lower at 7 and 28 days than M50 concrete. The compressive strengths of M50BS30, M50FA10BS20, and M50FA15BS15 concrete were around 23% lower than that of M50 concrete at 7 days, although at 28 days those of M50FA10BS20 and M50FA15BS15 concrete were 2.6% and 7.9% lower, respectively, and that of M50BS30 was similar. However, at 91 days, RFA concrete containing 30% blended cementitious materials had a higher compressive strength than M50 concrete. Compared with M50 concrete, the compressive strengths of M50BS30, M50FA10BS20, and M50FA15BS15 concrete increased by 9.8, 10.2, and 17.5%, respectively. Figure 1e shows that the concrete containing 45% blended cementitious materials. In addition, M50BS45 concrete had a 7-day compressive strength 24.1% lower than that of M50, a 28-day compressive strength 5.7% higher than that of M50 and similar to that of N, and a 91-day compressive strength 31.6% higher than M50 and 8.5% higher than N.M50BS45 was the only specimen that had a higher compressive strength than N at 91 days, and had the highest compressive strength at 91 days in this experiment. Compared with M50, the 7-day compressive strengths of M50FA15BS30 and M50FA22.5BS22.5 were 32.9% and 39.8% lower, and the 28-day compressive strengths were 9.8% and 18.4% lower, respectively. The compressive strength of every mixture proportion was higher than that of M50 at 91 days; M50BS45, M50FA15BS30, and M50FA22.5BS22.5 concrete were 31.6, 12.6, and 4.0% higher, respectively. Zhao et al. [69] researched concrete containing 30, 40, and 50% cementitious materials (fly ash and GGBS) and showed that the compressive strength of concrete decreased with the increase of cementitious materials content. In addition, the 28-day compressive strength increased with the increase of fly ash content at the same content of cementitious materials content. Gesoğlu et al. [70] found that concrete incorporating 10% fly ash and 10% GGBS had the highest compressive strength. Experimental results showed that adding cementitious materials to recycled aggregate concrete helps to increase its strength; especially M50BS45 and M50FA15BS15 exhibited higher and similar compressive strength than normal concrete, respectively. Therefore, it can be said that adding GGBS and fly ash can increase the compressive strength of RFA concrete, but the mixing ratio of GGBS and fly ash needs further study.

Figure 1f shows the compressive strength of RFA concrete with constant FA content (10% and 15%). In RFA concrete containing 10–15% FA, adding 15–30% GGBS resulted in the 7-day compressive strength being similar, the 28-day compressive strength increased by about 20%, and the 91-day compressive strength increased by about 15% compared with M50FA10, M50FA15 specimens. Therefore, adding GGBS to concrete containing FA can address the problem of low 28-day compressive strength of FA concrete and increase the 91-day compressive strength.

### 3.2. Drying Shrinkage

**Figure 2:**
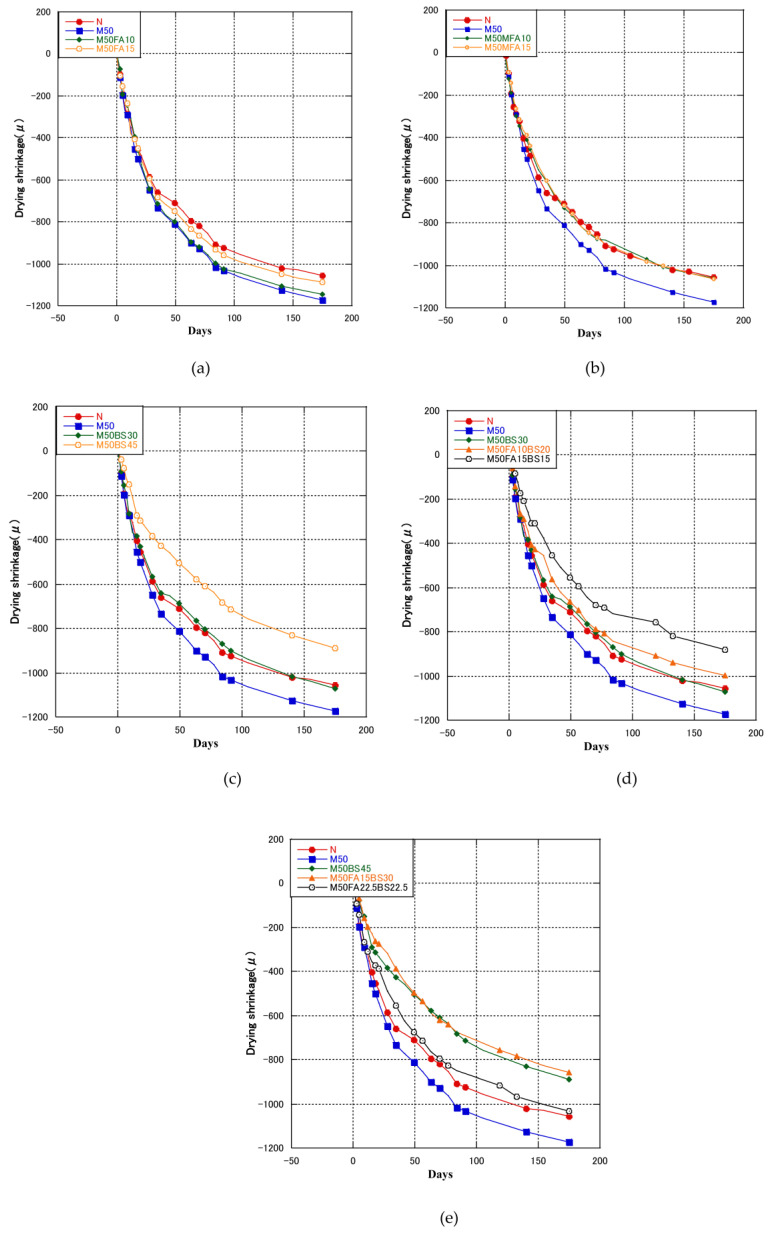
Drying shrinkage of concrete with cementitious materials.

Figure 2a shows the drying shrinkage of the FA specimens. The drying shrinkage of M50 developed quickly, diverged from the control concrete after 1 week, and was 10.85% higher after 182 days, Kirthika et al. [11] reported the similar results. It is common knowledge that the greatest cause of drying shrinkage is the water content of the concrete mixture [71]. The higher drying shrinkage of RFA concrete was due, in part, to RFA’s higher water absorption, and because a large amount of fines in the pores and gaps of coarse particles of RFA increased the paste volume of the concrete [2]. The drying shrinkage of RFA concrete containing FA was suppressed compared with M50; the drying shrinkages of M50FA10 and M50FA15 were 2.3% and 7.1% lower after 182 days, respectively. Thus, adding FA to RFA concrete decreased the drying shrinkage and the decrease was greater as the FA content increased. Figure 2b shows the drying shrinkage of the MFA specimens. Both M50MFA10 and M50MFA15 concrete had lower drying shrinkages than M50, which were similar to that of the control concrete. Compared with M50, the drying shrinkages of M50MFA10 and M50MFA15 were 9.5% and 9.3% lower at 182 days, respectively. Saha et al. [26], and Wang et al. [72] also reported similar results in that the drying shrinkage decreased with increasing fly ash content. Adding fly ash to concrete lowered the cement concentration and delayed the development of shrinkage, which caused a modest reduction in early drying shrinkage [31,73]. The results of pore structure in this experiment showed that fly ash modified the pore structure of concrete, which inhibited evaporation of water and reduced drying shrinkage.

Figure 2c shows the drying shrinkage of the GGBS specimens. GGBS reduced the drying shrinkage considerably. The drying shrinkage of M50BS30 at 182 days was 8.6% lower than that of M50, and was similar to that of the control concrete. Furthermore, at 182 days, the drying shrinkage of M50BS45 was the lowest, was 24% lower than that of M50 and 15.8% lower than that of the control concrete. Some studies also reported similar results [37,74,75], adding GGBS inhibited the development of drying shrinkage of concrete. Adding GGBS to concrete reduced the porosity and modified the pore structure of the concrete [33,76]. On the one hand, GGBS had a larger specific surface area than fly ash, and on the other hand, GGBS had a higher CaO content than fly ash, so the activity of GGBS was higher, and the inhibition effect on drying shrinkage was greater.

Figure 2d shows the drying shrinkage of the 30% blended cementitious materials specimens. The cementitious materials reduced the drying shrinkage, and the mixture of GGBS and FA had a greater effect. The drying shrinkage of M50FA10BS20 and M50FA15BS15 at 182 days was 14.8% and 24.7% lower than that of M50, and 5.6% and 16.5% lower than that of the control concrete, respectively. Figure 2e shows the drying shrinkage results of 45% blended cementitious material specimens. In contrast to the 30% blended cementitious material specimens, M50FA15BS30 had a similar 182-day drying shrinkage compared to M50BS45, whereas the 182-day shrinkage value of M50FA22.5BS22.5 was 16.2% higher than that of M50BS45. Therefore, for a cementitious materials content of 30–45%, the reduction in drying shrinkage was higher for GGBS mixed with FA, although FA contents higher than 15% increased the drying shrinkage.

Zhao et al. [69] reported that comparing concrete with the simultaneous addition of fly ash and GGBS to cement-only concrete, shrinkage was reduced by 15%. Weng et al. [77] studied concrete with binary cementitious materials (FA, GGBS) and showed that drying shrinkage of concrete was less with binary FA and GGBS than with GGBS alone. Due to GGBS’s fineness and hydration activity, which aid in the formation of a compact microstructure and stop water evaporating from the concrete, drying shrinkage of concrete is significantly reduced [77]. Adding fly ash or GGBS to concrete promoted the hydration of cement and modified the microstructure of concrete, lowing permeability of free water, so that drying shrinkage decreased significantly [69].

### 3.3. Accelerated Carbonation

**Figure 3:**
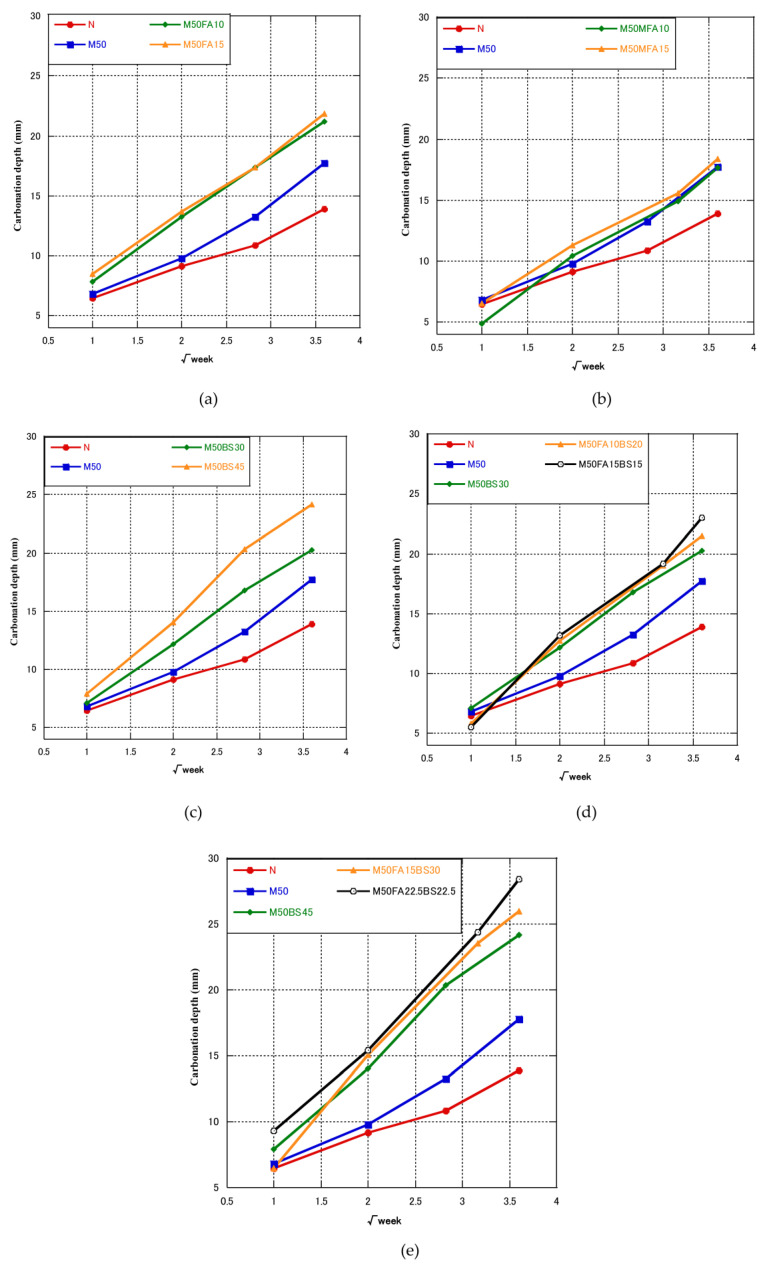
Carbonation depth of concrete with cementitious material.

The accelerated carbonation experiment results are shown in Figure 3a–e. The carbonation depth of M50 was about 27.8% greater than that of the normal concrete at 91 days, probably due to the higher porosity of the concrete. Therefore, RFA decreased the carbonation resistance. Some studies reported that the carbonation depth of RFA concrete likewise rises with the recycled aggregate replacement level [18,19] because concrete with RFA has higher porosity than normal concrete, which makes it easier for atmospheric CO_2_ to diffuse into concrete.

Figure 3a,b shows the results of the FA and MFA groups. Increasing the FA content increased the carbonation depth at 91 days, regardless of whether it was MFA or class II FA. Compared with the M50 concrete, the carbonation depths of M50FA10, M50FA15, M50MFA10, and M50MFA15 were 19.8, 22.9, 1.65, and 4.92% higher, respectively. Some research reported similar results and showed that the depth of carbonation increased as FA quantity increased [78,79,80]. This was due to the lower content of available Ca(OH)_2_ in fly ash concrete compared to normal concrete [81,82], which resulted in faster carbonation of the C-S-H bond [83].

Figure 3c show the results of GGBS group. The results were similar to concrete containing FA. Increasing the GGBS content increased the carbonation depth at 91 days. Compared with the M50 concrete, the carbonation depths of M50BS30 and M50BS45 were 14.2% and 31.6% higher, respectively. It was reported that with up to 20% GGBS content, the carbonation of GGBS concrete was similar to that of normal concrete, while with a GGBS content over 20%, carbonation depth increased with the increase of GGBS content [84]. Sulapha et al. [85] also reported that concrete with GGBS exhibited lower resistance to carbonation than conventional concrete.

Figure 3d,e shows the results of the blended admixture (FA, GGBS) group. Increasing the blended admixture content (FA and GGBS) increased the carbonation depth at 91 days. In concrete containing 30% and 45% blend admixtures, increasing the FA content increased the carbonation depth, and M50FA22.5BS22.5 had the highest carbonation depth. Therefore, FA had a greater effect on carbonation than GGBS, although after 7 days, specimens containing FA had a smaller carbonation depth. Jones et al. [86] studied concrete containing ternary binders, and showed that compared to regular concrete, concrete incorporating GGBS and fly ash showed noticeably greater rates of rapid carbonation. On average, carbonation depths for the concrete made with GGBS and fly ash mixes were 2.5 times greater than those for regular concrete, and as the cement replacement level was raised, carbonation rates increased [86].

**Figure 4:**
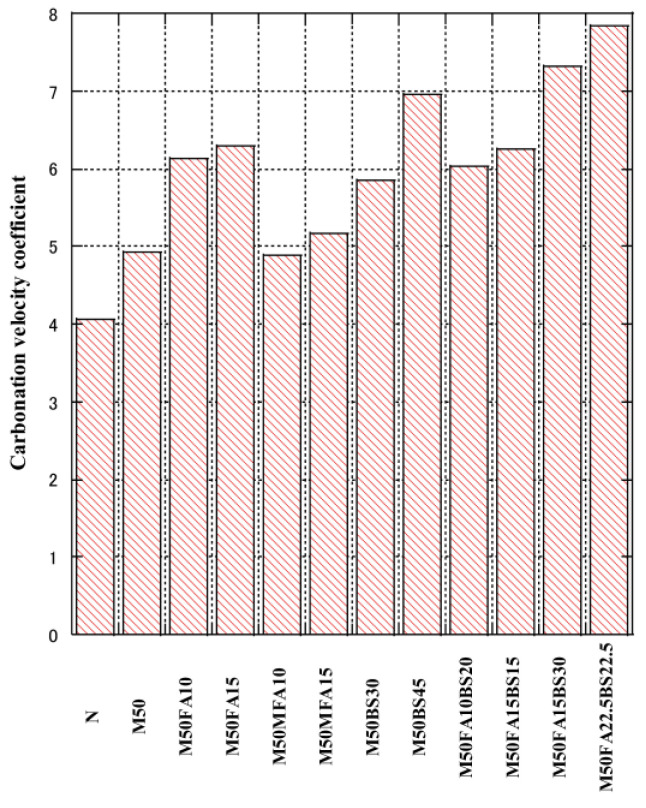
Carbonation velocity coefficient.

Figure 4 shows the carbonation velocity coefficients. The incorporation of FA, GGBS, and RFA increased the carbonation velocity coefficient. In the RFA concrete containing 30% and 45% blended admixtures, the carbonation depth increased with the FA content. Generally, increasing the content of mineral admixture increases the carbonation depth of concrete. This is mainly because the pozzolanic reaction consumes a large amount of Ca(OH)_2_, resulting in a decrease in the pH of the concrete [87]. When the FA content was constant, adding GGBS to the concrete increased the carbonization velocity coefficient.

When the total FA and GGBS content was constant, increasing the FA content increased the carbonation velocity coefficient.

### 3.4. Pore Structure

**Figure 5:**
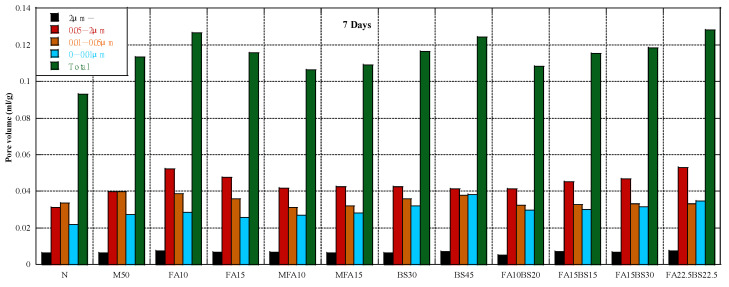
Pore structure at 7 days.

**Figure 6:**
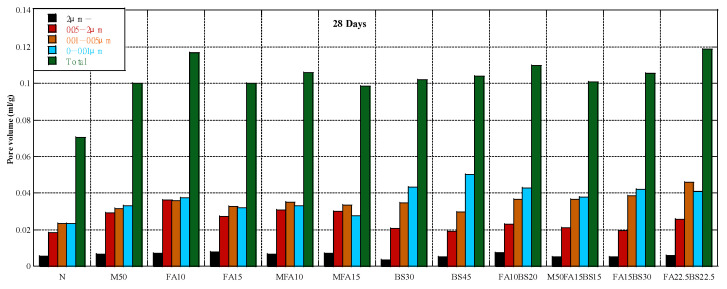
Pore structure at 28 days.

**Figure 7:**
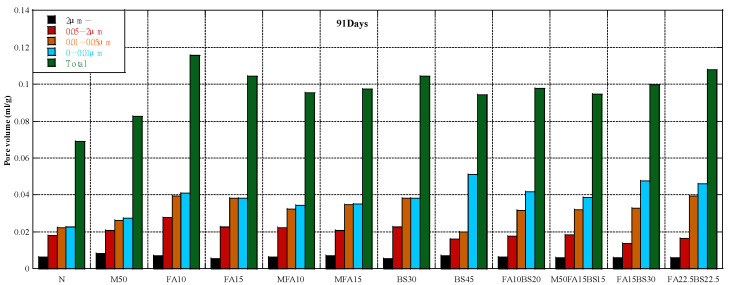
Pore structure at 91 days.

Figure 5 shows the cumulative pore volume at 7 days. The concrete containing RFA had a higher pore volume, especially for pore diameters of 0.05–2 µm. The concrete containing class II FA had a higher pore volume than M50 concrete, whereas the concrete containing MFA had a lower pore volume. Thus, the properties of FA strongly affect the pore volume of concrete. The concrete containing GGBS had a higher pore volume than M50 concrete, and the pore volume increased with the GGBS content. The concrete containing the blended admixtures had a lower pore volume when the admixture content was 30% compared with concrete containing only GGBS, and the ratio of FA to GGBS of 1:2 was better than 1:1. Figure 6 shows the cumulative pore volume at 28 days. The effect of RFA on the pore volume of concrete was similar to the effect at 7 days, and the concrete containing RFA had a higher pore volume than the control concrete. The pore volume at 28 days was much lower than that at 7 days due to the hydration of the cement and cementitious materials. The concrete containing fly ash had different results; that containing class II FA had a higher volume than the control concrete, whereas that containing MFA had a lower pore volume. For concrete containing GGBS, the change in cumulative pore volume was negligible, but the pore volume between 0.05 and 2 µm was considerably lower. For concrete containing the blended admixture, concrete containing FA and GGBS had a higher pore volume than concrete containing only GGBS, especially for pore diameters of 0.01–0.05 µm. When the cementitious materials content was constant, the pore volume increased with the FA content. Figure 7 shows the cumulative pore volume at 91 days. The pore volume of M50 was higher than that in the control concrete. The pore volume of RFA concrete containing FA was larger than that in the M50 concrete, but the volume of pores between 0.05 and 2 µm did not change much, mainly due to the increase in the volume of pores less than 0.05 µm, regardless of whether the FA was class II FA or MFA. This may be due to the pozzolanic reaction and the tiny aggregate effect of FA [71]. Poon et al. [88] also reported that replacing cement with fly ash increased porosity but decreased average pore size of the pastes. Other studies reported similar results [89,90]. The total pore volume of concrete containing GGBS was larger than that of M50 concrete, but the 0.05–2 µm pore volume was smaller than that of M50 concrete, mainly due to the increase in the volume of pores less than 0.05 µm.

The pore volume of concrete containing 30% cementitious materials did not change substantially, whereas that containing 45% cementitious materials was different. The total pore volume of concrete containing the blended admixture was larger than that containing only GGBS; while the volume of the pores at 0.05–2 µm did not change much, the volume of the pores at 0.01–0.05 µm increased. The experimental results prove that the activity of pozzolanic reaction of GGBS is greater than that of fly ash, so the pore volume of concrete mixed with GGBS only had a higher pore volume at pore diameters of less than 0.01 µm. Furthermore, we found that the incorporation of GGBS decreased the volume of pores with a diameter greater than 0.01, and the incorporation of fly ash had little effect on the volume of pores. with a diameter of 0.05–2µm but increased the volume of pores with a diameter of 0.01–0.05µm.

Based on MIP-determined trials, Mindess et al. [91] defined capillary pores as being greater than 0.01 μm, and gel pores as being less than 0.01 μm. PK Mehta [92] analyzed pore size in four ranges: less than 4.5 nm, 4.5–50 nm, 50–100 nm, and greater than 100 nm. Wu and Lian [93] examined four ranges of pores: pores under 20 nm, between 20 and 50 nm, 50 and 200 nm and those over 200 nm. It was reported that the strength of mortar was mainly affected by capillary pores, and the durability was mainly affected by gel pores [94].

**Figure 8:**
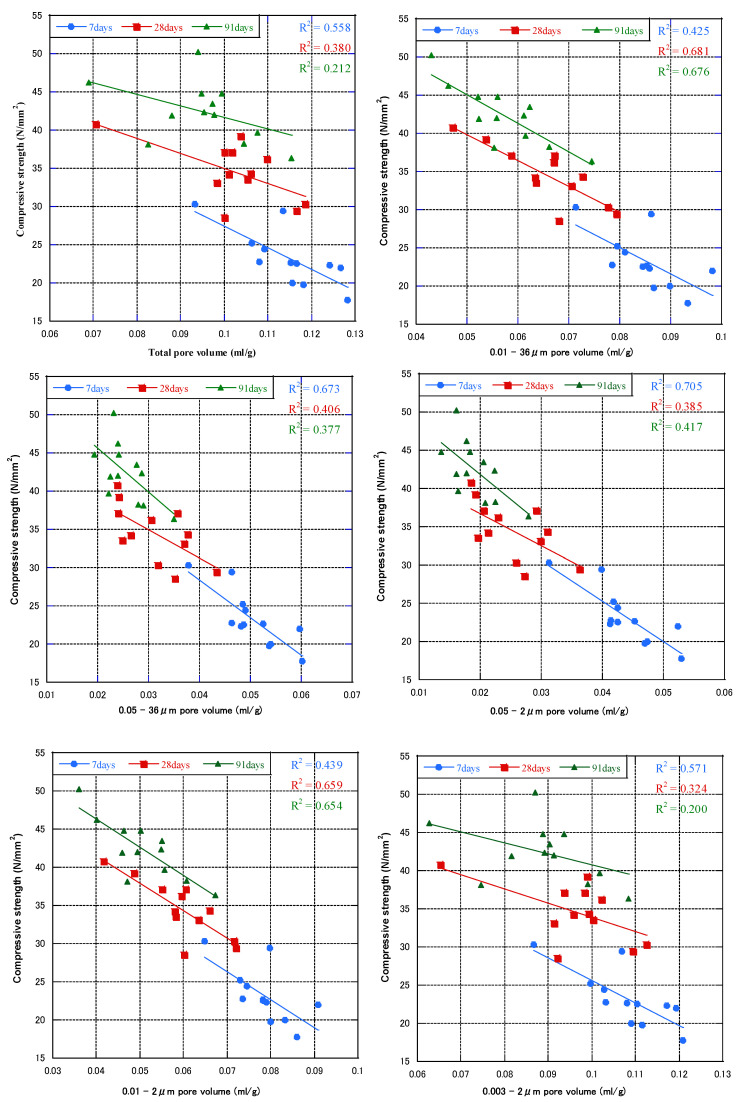
Relationship between pore volume and compressive strength at each age.

**Figure 9:**
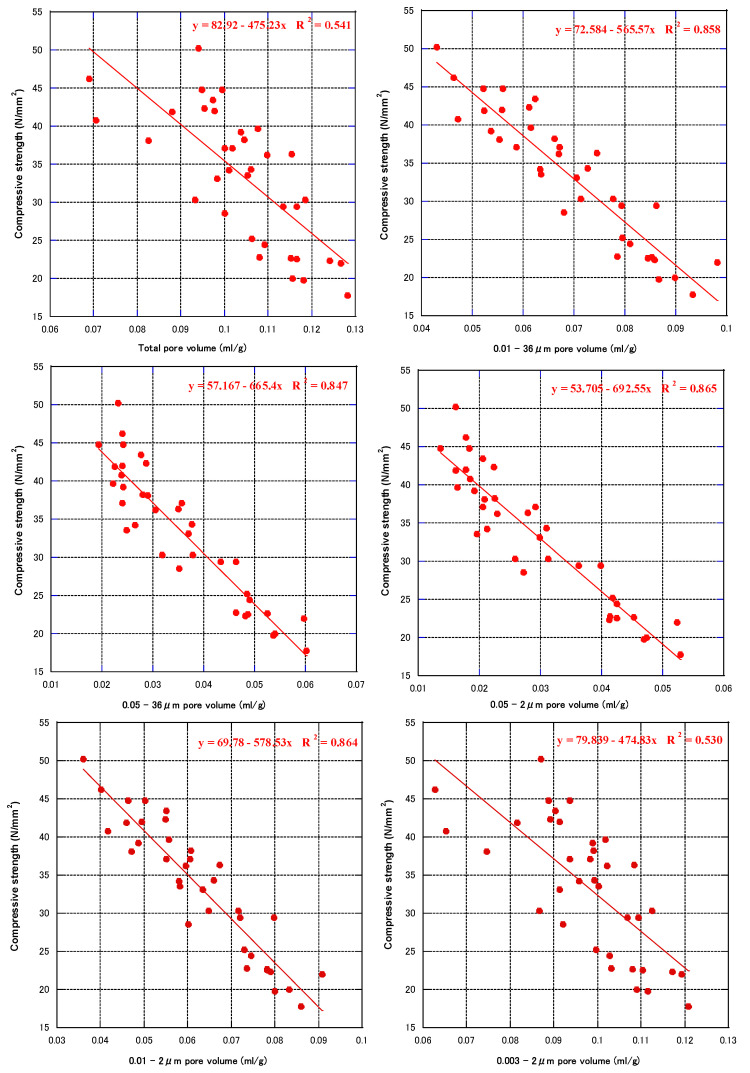
Relationship between pore volume and compressive strength (all mixes and ages).

Based on the relationship between pore volume and pore diameter, we analyzed the correlation between the compressive strength of concrete at each age and the total pore volumes of 0.01–36, 0.05–36, 0.003–2, 0.01–2, and 0.05–2 μm. Figure 8 shows the relationship between compressive strength and cumulative pore volume at each age (all mix proportions). In all pore size ranges, the compressive strength tended to increase as the pore volume decreased. For the 28-day and 91-day compressive strength, the correlation coefficients (R^2^) for the 0.01–36 µm pore volume were the highest. However, the 7-day compressive strength had a good correlation with the 0.05–2 μm pore volume. In Figure 9, regardless of the age and mix proportions of the specimens, all the compressive strength and cumulative pore volumes obtained were fitted. The compressive strength had a low correlation with the total pore volume and pore volume of 0.003–2 μm. Furthermore, compressive strength was linearly related to pore volumes larger than 0.01 or 0.05 μm. This showed that pores with a diameter of less than 0.01 μm had no effect on the compressive strength of concrete, pores with a diameter of 0.01–0.05 μm had weak effect on the compressive strength of concrete, and pores with a diameter greater than 0.05 μm had a greater effect on the strength of concrete. The correlation coefficient between the 0.05–2 μm pore volume and the compressive strength had the largest R^2^ of 0.87. Therefore, for the compressive strength, pores with a diameter greater than 0.05 μm can be termed harmful pores, 0.01–0.05 μm small harmful pores, and less than 0.01 μm harmless pores.

**Figure 10:**
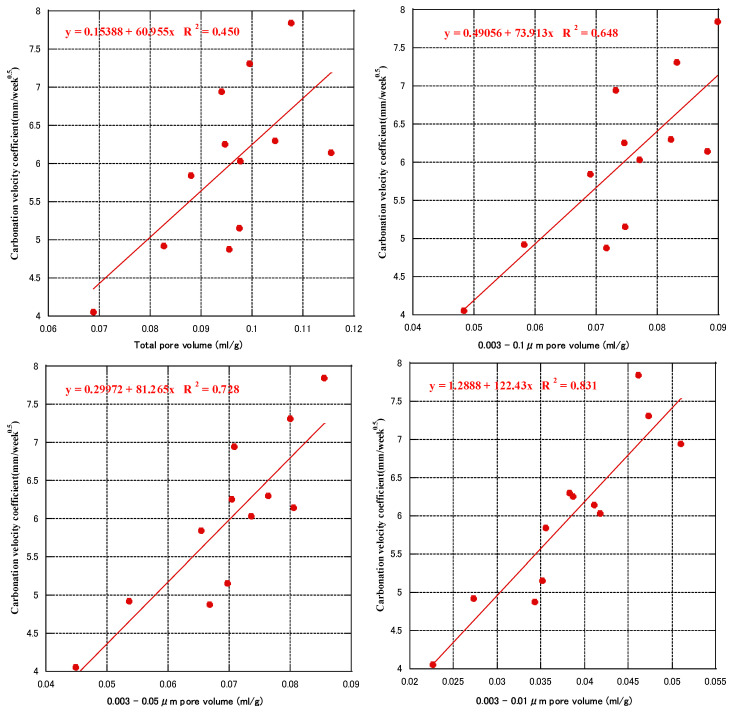
Relationship between cumulative pore volume and carbonation velocity coefficient.

Figure 10 shows the correlation between carbonation velocity coefficient and cumulative pore volume obtained from the MIP tests at 91 days (total pore volumes, pore volumes of 0.003–0.1, 0.003–0.05, and 0.003–0.01 μm), respectively. The correlation between cumulative pore volume and carbonation velocity coefficient increased gradually with the decrease of pore diameter. The correlation between the carbonation velocity coefficient and 0.003–0.01 μm pore volume was the highest, with R^2^ of 0.83. Thus, the carbonation velocity coefficient of concrete was linearly related to the volume of pores with diameters less than 0.01 µm.

## 4. Conclusions

1. Adding fly ash and GGBS to RFA concrete increased its compressive strength. M50BS45 and M50FA15BS15 exhibited similar 91-day compressive strengths with normal concrete. Therefore, the compressive strength of RFA concrete can be effectively improved by the use of cementitious materials.
2. Replacement of cement with fly ash or GGBS significantly decreased the drying shrinkage of the RFA concrete. The drying shrinkage of all specimens in this experiment reached the level of ordinary concrete, and even lower than that of ordinary concrete. M50FA15BS15 showed 16.5% lower drying shrinkage, M50BS45 and M50FA15BS30 showed around 25% lower drying shrinkage than normal concrete.
3. Increasing the cementitious (fly ash and GGBS) materials content decreased the carbonation resistance of RFA concrete. Fly ash had a greater effect on carbonation than GGBS.
4. Incorporating FA or GGBS into concrete modified the pore structure of concrete, and reduced the volume of capillaries larger than 0.05 μm. In addition, the compressive strength was mainly affected by capillary pores (greater than 0.01 μm or 0.05 μm), and the carbonation was mainly affected by gel pores (less than 0.01 μm).
5. For compressive strength, pores with a diameter greater than 0.05 μm are considered harmful pores, 0.01–0.05 μm are considered small harmful pores, and less than 0.01 μm are considered harmless pores.

## Figures and Tables

**Table 1:** Properties of fine and coarse aggregates.

Property	Coarse Aggregate	Sea Sand	RFA	JIS A5022 (M)
Oven-dried density (g/cm^3^)	2.69	2.59	2.37	>2.2
Fineness modulus	6.9	2.41	2.58	_
Water absorption (%)	1.41	1.04	6.86	<7.0
Void content (%)	43.3	38.8	32.6	_

**Table 2:** Properties of the cement, FA, MFA, and GGBS.

	FA	MFA	GGBS	Cement
SiO_2_ (%)	53.8	62.4	32.7	21.5
Al_2_O_3_ (%)	13.5	17.6	13.4	5.4
Fe_2_O_3_ (%)	13	8.7	0.5	3.0
CaO (%)	8.99	2.3	41.6	64.9
SO_3_ (%)	0.49	_	6.9	1.4
MgO (%)	1.48	1.32	0.3	2.1
Loss on ignition (%)	2.1	1.2	0.6	0.8
Density (g/cm^3^)	2.31	2.18	2.91	3.16
Blaine specific area (cm^2^/g)	3270	5480	4100	3000

**Table 3:** Mix proportions.

Type					Unit Mass (kg/m³)				
	W/B	W	C	FA	MFA	GGBS	S	RFA	G
N	0.55	180	327	0	0	0	832	0	945
M50	0.55	180	327	0	0	0	416	379	945
M50FA10	0.55	180	294	33	0	0	411	375	945
M50FA15	0.55	180	278	49	0	0	409	372	945
M50MFA10	0.55	180	294	0	33	0	411	375	945
M50MFA15	0.55	180	278	0	49	0	409	372	945
M50BS30	0.55	180	229	0	0	98	413	376	945
M50BS45	0.55	180	180	0	0	147	411	375	945
M50FA10BS20	0.55	180	229	33	0	65	409	373	945
M50FA15BS15	0.55	180	229	49	0	49	407	371	945
M50FA15BS30	0.55	180	180	49	0	98	405	369	945
M50FA22.5BS22.5	0.55	180	180	74	0	74	402	367	945

## Data Availability

The data presented in this study are available on request from the corresponding author. The data are not publicly available due to privacy restrictions.
